# Mesenchymal stem cell therapy for laryngotracheal stenosis: A systematic review of preclinical studies

**DOI:** 10.1371/journal.pone.0185283

**Published:** 2017-09-21

**Authors:** Kathrine Kronberg Jakobsen, Christian Grønhøj, David H. Jensen, Anne Fischer-Nielsen, Thomas Hjuler, Christian von Buchwald

**Affiliations:** 1 Department of Otorhinolaryngology, Head and Neck Surgery and Audiology, Rigshospitalet, University of Copenhagen, Copenhagen, Denmark; 2 Cell Therapy Facility, Blood Bank, Department of Clinical Immunology, Rigshospitalet, University of Copenhagen, Copenhagen, Denmark; Università degli Studi della Campania "Luigi Vanvitelli", ITALY

## Abstract

**Background:**

Laryngotracheal stenosis (LTS) can be either congenital or acquired. Laryngeal stenosis is most often encountered after prolonged intubation. The mechanism for stenosis following intubation is believed to be hypertrophic scarring. Mesenchymal stem cells (MSCs) therapy has shown promising results in regenerative medicine. We aimed to systematically review the literature on MSC therapy for stenosis of the conductive airways.

**Methods:**

PubMed, EMBASE, Google Scholar and the Cochrane Library were systematically searched from January 1980–January 2017 with the purpose of identifying all studies addressing the effect of MSC therapy on the airway. We assessed effect on inflammation, fibrosis, and MSC as a component in tissue engineering for treating defects in the airway.

**Results:**

We identified eleven studies (n = 256 animals) from eight countries evaluating the effect of MSCs as a regenerative therapy in the upper airways. The studies indicate that MSC therapy may lead to a more constructive inflammatory response as well as support tissue regeneration.

**Conclusion:**

There may be a favorable effect of MSCs in inhibiting inflammation and as a component in tissue engineering. Given the heterogeneous nature of the included animal studies, any clear conclusion regarding the effect of tracheal stenosis in human subjects cannot be drawn. The included preclinical studies are however encouraging for further research.

## Introduction

### Characteristics of laryngotracheal stenosis

Laryngotracheal stenosis (LTS) is a broad term encompassing narrowing of the airway at the level of the larynx, subglottis, or trachea. Laryngotracheal stenosis (LTS) is a rare but severe condition. Besides the functional impairments as a result of airway obstruction, stenosis of the airways leads to considerable morbidity and mortality. [1,2] The main cause of LTS is intubation. [3] Current options for treating stenosis involve endoscopic dilation, laser surgery, laryngotracheal reconstruction, or life-long tracheostomy, but are often suboptimal as new scar tissue frequently develops leading to restenosis. [3,4] It has been suggested that LTS develops because of altered fibroblast responsiveness to anti-fibrotic signals during mucosal repair leading to excessive production of fibrosis. [5] Furthermore, there seems to be an altered inflammatory response leading to hypertrophic scars and a correlation between early inflammatory reaction to injury of the mucosa and the degree of scarring. [6]

The initial, preferred and less invasive method of treatment is endoscopic dilation, but this method has only proven success rates in paediatric patients of 64%; not incorporating the need for repetitive treatments due to re-stenosis and accompanying morbidity. [3,4] Alternatively, laryngotracheal reconstruction is required for severe stenosis. [3,7] This procedure holds high success rate but entails severe risks and subsequent morbidity. [6]

### Mesenchymal stem cells

Mesenchymal stem cells (MSCs) are adult multipotent stem cells characterized by their adherence to plastic, their surface antigen expression, and their ability to differentiate into various connective tissue lineages including adipogenic, chondrogenic, myogenic and osteogenic cells. [8,9]

MSCs have during the last decades received extensive interest due to their potential therapeutic use as treatment for multiple diseases. [10] MSCs have shown anti-inflammatory and immunosuppressive properties, an ability to migrate to the exact site of injury, and a capacity to secret soluble factors crucial for cell survival and proliferation with minimal side effects. [10,11] Furthermore, MSCs currently indicate promising results in regenerative medicine. [10] In addition, MSCs are easily accessible for isolation most commonly from the bone marrow (BM-MSC) or adipose tissue (ASC) and have shown great expansion potential supporting the prospective of MSCs as a therapeutic agent. It has been shown that ASC increase wound healing with less scarring in skin ulcers [12] and the prospect of using ASC for therapeutic purpose in treating LTS is promising.

This study systematically evaluated the literature on the effects of MSCs on the conductive airway. We aimed to clarify the potential of MSCs in the conducting airways in regard to potentially treating stenosis and to evaluate their potential in a future human trial.

## Materials and methods

This systematic review was conducted with reference to the Preferred Reporting Items for Systematic Reviews (PRISMA) statement. [13]

### Systematic literature search and eligibility criteria

In January 2017, one author (KKJ) systematically searched the PubMed, EMBASE, Google Scholar, Cochrane Library, and clinicaltrials.org for articles in the English and Scandinavian languages. We included studies evaluating the effect of exogenous supplied MSC on the conductive airway in animals regardless of the publication date. Only studies concerning the conductive airways (trachea to the bronchioles) were included. Studies concerning the effector-mechanisms by which MSCs exert their properties were excluded as were studies evaluating the effect of MSCs on the airway in combination with other therapeutic regimes. Finally, studies concerning the effect of MSCs on the alveolar function, lung parenchyma and vascular bed were excluded. Due to conflict of interests the study done by Macchiarini, P. et al. on clinical transplantation of a tissue-engineered airway was excluded.

The following keywords (MeSH terms included in PubMed) were used: mesenchymal stromal cell, or MSC, or mesenchymal stem cell, adipose derived stromal cells, or adsc preadipocytes, or processed lipoaspirate cells, or stromal vascular fraction cells and Airways when searching PubMed and Embase. In Google Scholar, clinicaltrials.org and Cochrane Library the search were performed with the keywords “stem cell” and “airways”.

The following data were extracted from the included studies: study design, study participants, graft donor, source of graft, intervention, control groups, origin of stem cell, statistical tests.

## Results

The electronic searches identified 251 potentially eligible studies, of which eleven studies (256 animals) met the inclusion criteria (S1 Fig). All studies concerned the use of MSCs as a regenerative modality in the treatment of *upper* airway disease.

It was not possible to perform a meta-analysis due to the great variability in species, source of the stem cell, evaluation time, disease model, intervention and statistical tests across the studies.

### The effect of MSCs on inflammation in the airway

Five studies (n = 153) evaluated the effect of MSCs on inflammation in the airways, (Table 1). [14–18] All studies used a preclinical model of induced asthma with either ovalbumin or toluene diisocyanate (TDI) as an inflammatory sensitizer (Table 1). [14–18]

**Table 1:** Overview of studies: MSCs and inflammation in the airways.

Author (Year) [reference]	Animal	Origin of MSCs	Intervention	Group(s)	Focus of interest	Statistic test	Study design
Firinci F. et al (2011) [17]	Mice, 6-8-week-old, weighing 18 to 20 g. N = 72	Allogenic from bone marrow from mice	Sensitized via intraperitoneal of chicken egg albumin with alum as an adjuvant. After the sensitization, the mice in study groups 2 and 3 were exposed to aerosolized ovalbumin for 30 min per day on three days a week for eight weeks, beginning from the 21st day of the study. MSCs were administered following the OVA nebulization.	Control group Ovalbumin induced asthma only Ovalbumin induced asthma + MSCs MSCs only	Examination of the efficacy of MSCs on lung histopathology especially remodeling in a murine model of chronic asthma	The comparisons between all groups were conducted by using Kruskal–Wallis method. When differences were statistically signicant, Mann–Whitney U test was used for group comparisons.	Controlled trial. Investigators were blinded to the treatment groups when interpreting the analyzes.
Mohammadian M. et al. (2016) [16]	Mice, 6–8 weeks old. N = 14	Allogenic MSC derived from bone marrow from mice	Sensitization by intraperitoneal injection of ovalbumin and aluminium hydroxid and one week after exposed to aerosolized OVA for 30 min per day on three days a week for eight weeks. MSC were administered on the last week of OVA challenge.	Control group, not sensitized Asthma group Asthma+MSC group	Effect of MSCs on lung pathology and inflammation in ovalbumin-induced asthmatic mice.	Comparison between groups (control, asthma and asthma+MMSC) were preformed using analyze with analysis of variance (ANOVA) followed by the Turkey test.	Randomized, controlled trial.
Ogulur I et al. (2014) [15]	Mice, 6–8 weeks old. N = 14	Allogenic MSC derived from bone marrow from mice	Intraperitoneally sensitized with chicken egg albumin and exposed to aerolized OVA. MSCs were administered intravenously just after the last nebulization of OVA.	Control group exposed to normal saline i.p. and then exposed to aerolized PBS OVA OVA + MSC PBS + MSC groups	Evaluation of the effect of MSCs on airway remodeling and inflammation in a ovalbumin-induced mouse model of chronic asthma	The difference between groups was analyzed with one-way ANOVA test.	Controlled trial. Blinded investigators.
Sun YQ. et al. (2012) [14]	BALB/c mice, 4–6 weeks of age. N = 35	Xenogenic MSCs from human bone marrow	Sensitized by intraperitoneal injection of ovalbumin in phosphate-buffered saline (PBS) on days 1, 3, 5, 7, 9, 11, and 13. From days 21 to 27 challenged daily with aerosolized OVA. Subsequently, mice were intranasally infused with OVA. MSCs were injected via the tail vein at day 0 before the sensitization and on day 20 after the sensitization but before the challenge.	Sensitization/challenge/injection with PBS/PBS/PBS Sensitization/challenge/injection with Naïve/Naïve/iPSC-MSCs Sensitization/challenge/injection with OVA/OVA/PBS Sensitization/challenge/injection with OVA/OVA/iMR90-iPSC-MSCs Sensitization/challenge/injection with OVA/OVA/N1-iPSCS-MSCs Sensitization/challenge/injection with OVA/OVA/BM-MSCs Sensitization/challenge/injection with iPSC-MSCs /OVA/OVA	Evaluation of the effect of systemic administration of BM-MSCs and iPSC-MSCs on allergic inflammation in ovalbumin-induced allergic inflammation in upper and lower airways.	One-way analysis of variance followed by a Student-Newman-Keuls test for multiple comparisons of the data with Gaussian distribution. A Kruskal–Wallis rank sum test followed by a Mann–Whitney U test was performed for two-group comparisons of the data with abnormal distribution.	Controlled trial. Mice were subjected to a single-blind observation by examiners who had no knowledge of the experimental groups.
Lee SH. et al. (2011) [18]	BALB/c mice, 6 weeks old. N = 18	Xenogenic MSCs from bone marrow of rats	Sensitized intranasally by Toluene Diisocyanate (TDI). Afterwards animals were challenged with TDI through ultrasonic nebulization. MSCs were injected intravenously one day before TDI challenge.	Sham TDI TDI + MSCs	Investigated of the effects of BMDMSCs in airway remodeling and inflammation in an experimental toluene diisocyanate(TDI)-induced asthma model.	Continuous data were compared using the Kruskal–Wallis test. If differ- ences were found to be significant, the Mann–Whitney U-test was applied to compare differences between two samples.	Controlled trial.

Three of the studies (n = 100) administered MSCs *after* sensitization [15,17] while two studies (n = 53) [14,18] administered the MSCs *before* the sensitization. [18] All studies used BM-MSCs and administrated the MSCs via intravenous injection.

All studies could demonstrate a reduction in inflammation in the MSC treated group compared to the control group (Table 1). This was either measured as a significant decrease in the amount of inflammatory cells peribronchially [14,15,18] a reduction in the total white blood cell count, or a significant reduction in neutrophiles and eosinophils in blood samples [16,18]. Finally two studies demonstrated a significant reduction in the level of pro-inflammatory cytokines in the circulation in the MSC treated group compared to the control group. [14,17] In three of the studies the MSCs group was also demonstrated to have a significant reduction in goblet cell hyperplasia compared to the control group. [15,16,18]

Furthermore, two studies demonstrated a significant reduction in the thickness of the epithelium, the subepithelial smooth muscle layers, and the basement membrane in the upper airways. [15,17]

### The effect of MSCs on fibrosis in the airways

Two of the above studies (n = 32) also addressed MSCs effect on fibrosis and collagen deposit in the upper airway (Table 1). [16,18]

One study on induced asthma was not able to demonstrate a significant change in fibrosis after administration of MSCs, evaluated by the amount of collagen deposition. [16] Another study on induced asthma [18] demonstrated an increase in collagen deposition following the sensitizer. However, administering MSCs caused that the amount of collagen deposition, only reached the level in the sham group.

### The use of MSCs in tissue repair

Six studies (n = 103) investigated the use of MSCs as a means to induce tissue repair, (Table 2). [7,19–23]

**Table 2:** Study results of the effect of MSCs on tissue engineering.

Author (Year) [reference]	Animal	Origin of MSCs	Intervention	Group(s)	Focus of interest	Statistic test	Study design
Ott LM. et al. (2015) [7]	Rabbits, 7–8 ib. N = 30	Allogeneic MSCs from rabbit bone marrow	Elliptical defect was made in the trachea ~ 2 cm below the cricoid cartilage. The defect was patched with a biomaterial scaffold patch. The scaffold was either encapsulated with TGF-β3, seeded with BMSCs, or was scaffold-only.	Scaffold-only Scaffold + TGF- β3 Scaffold + MSC	Evaluation of a scaffold for patch-type tracheal defects and evaluation of the benefits of adding cells or growth factor.	Statistical analysis was preformed using one-way ANOVA and Tukey’s post hoc analysis.	Controlled trial. The histological sections were scored blind by a pathologist
Gray FL. et al. (2012) [19]	Fetal lambs with tracheal defect. N = 13	Autologous MSCs derived from amniotic fluid	Complete tracheal segments from adult rabbits were decellularized. Each decellularized airway scaffold was then seeded with labeled aMSCs from only 1 donor fetus. Fetal lambs (N = 13) underwent an anterior longitudinal cervicotomy followed by a complete segmental resection of the native trachea, with its cranial border at least 4 tracheal rings below the cricoid.	Tracheal defect was repaired with a decellularized leporine tracheal segment Tracheal defect was repaired with an identical graft seeded with expanded/labeled autologous aMSCs	Examination of possibilities for tracheal repair with either a decellularized airway scaffold or a graft engineered from autologous amniotic mesenchymal stem cells (aMSCs).	Statistical comparisons were by 2-way repeated- measures analysis of variance and the Fisher's Exact test, as appropriate	Controlled trial.
Mendez JJ. et al. (2014) [21]	Rats, 3–5 months old. N = unknown	Xenogenic MSCs from human bone marrow and human adipose tissue	Rat lungs were decellularized, followed by seeding the matrix with hBM-MSCs or hAT-MSC	Decellularized rat lungs seeded with MSCs from bone marrow Decellularized rat lungs seeded with MSCs from adipose tissue	Ability of hBM-MSCs and hAT-MSCs to repopulate acellular rodent lung tissue.	T-tests were performed to evaluate whether two groups were significantly different from each other	Cohorte study
Daly AM. et al. (2012) [22]	Mice, 8–24 weeks. Adult rats, 16 weeks. (N = unknown)	Allogeneic MSCs from bone marrow of adult male mice	Lungs were decellularized and afterwards reseeded with MSCs.	Decellularized rat lungs seeded with MSCs from bone marrow	Examination of the structural features of the decellularized lung and examination of the growth and differentiation of MSCs on decellularized lung tissue.	Differences between results were assessed by unpaired t-test. Statistical analyzed by one-way analysis of variance (ANOVA) with Bonferroni post hoc analysis and post-test Dunnett or Newman-Keuls multiple comparison analyses	Cohorte study
Serikov VB. et al. (2007) [20]	Mice, 3-5-week-old. N = 30	Allogeneic MSCs from bone marrow of adult male mice	Animals received sublethal dose of whole-body irradiation the day before they were infused with MSC into the jugular vein. One month after MSC transplantation either naphthalene IP in corn oil or corn oil without naphthalene (control) where administered.	MSC transplantation + no naphthalene MSCs + naphthalene MSCs intratracheal No intervention Control	Examination of the participation of BM-cells in the process of airway epithelial restoration after naphthalene-induced injury.	Statistical analysis was performed using Mann–Whitney–Wilcoxon test	Controlled trial.
Go, T. et al. (2010) [23]	Pigs, 65 +/- 4 kg. N = 30	Autologous MSCs derived from bone marrow	Trachea was retrieved from 10 donors. BM-MSCs and mucosal epithelial cells were obtained from 20 intended recipients. 6 cm of the trachea in the recipients were replaced. Animals were observed for a maximum of 60 days. Tracheas were harvested and evaluated postmortem.	6 cm of trachea was replaced with: decellularized matrix 6 cm of trachea was replaced with: decellularized matrix and external, autologous MSC-derived chondrocytes 6 cm of trachea was replaced with: decellularized matrix with internal, autologous epithelial cells 6 cm of trachea was replaced with: decellularized matrix with both types	Examination of the relative contribution of epithelial cells and MSC-derived chondrocytes to the survival of tissue-engineered airway transplants in pigs.	Continuous variables were compared by using the independent-samples t test	Randomized, controlled trial.

One study (n = 30) [20], investigated the participation of MSCs in the recovery of injured airway epithelium. Naphthalene was used to induce lung damage one month after MSC transplantation into the jugular vein. It was observed that BM-MSCs could adhere to the airways and form patches of epithelial lining in the conductive airways. [20]

Two studies (n = unknown) evaluated the repopulation of decellularized lung scaffold with MSCs for subsequent clinical transplantation. [21,22] Mendez JJ. *et al*. [21] showed attachment to the airways when using MSCs derived from adipose tissue. In regard to epithelization, Mendez JJ. *et al*. showed that MSCs from both bone marrow and adipose tissue underwent epithelial cell differentiation.

This contrasts to Daly AM. *et al*. [22] that repopulated their scaffolds with BM-MSCs but their cells did not differentiate into pseudostratified epithelia.

Three studies (n = 73) evaluated the repair of a tracheal defect by surgically replacing the damaged area with a tracheal graft or a scaffold. [7,19,23] Ott LM. *et al*. [7] and Gray FL. *et al*. [19] used patch-type decellularized scaffold to repair a tracheal defect and investigated if seeding of MSCs onto the scaffold would be beneficial for the tracheal repair, measured by survival rate, severity of the stenosis, the epithelialization, and histology of the scaffold. Go, T. *et al*. (n = 30) [23] investigated the engineering of a functional long-segment graft and replaced six cm of the trachea with a tracheal graft. The tracheal defects were made on the lamb fetus, and after the defect was repaired the fetus was returned to the uterine cavity.

Ott LM. *et al*. found that the survival rate was higher in the BM-MSC group compared to the scaffold-only group and had a higher level of immature cartilage which could indicate that BM-MSCs contribute to cartilage formation.

In regards to cross section area and preventing stenosis no clear results exist. Ott LM. *et al*. observed a larger cross section area in the scaffold-only group compared with the scaffold seeded with BM-MSC. Gray FL. *et al*. did not show a significant difference in cross section area between the two groups. Gray FL. *et al*. argued that the scaffolds were made from tracheal segments of rabbits and were thus not originally programmed to grow or dilate. Go T. *et al*. showed that MSC-derived chondrocytes seeded on the external surface resulted in less stenosis of the tracheal graft. Go T. *et al*. [23] continued showing the beneficial effect of epithelialization which help prevent bacterial/fungal contamination.

## Discussion

To the best of our knowledge, this study is the first systematic review to evaluate the effect of mesenchymal stem cells in the conducting airway and its therapeutic preclinical potential for treating laryngotracheal stenosis. As we identified no studies specifically evaluating the effect of MSCs in regards to treating stenosis by injecting stem cells into the airways, this review highlights the effects of MSCs on the airways in a general setting. The goal of the study was to investigate the effects of MSCs on the airway as a preclinical study to evaluate if it could be possible to treat stenosis by injection of stem cells to the airway. The result of our study highlighted three main areas respectively the impact on inflammation, on fibrosis and in tissue repair.

The studies included in this review differed in regards to origin of MSCs, the dose of MSCs, the study design, the animal model and the disease model. Collectively, both mice, rats, rabbits, pigs and lambs were used as experimental models. MSCs were derived from bone marrow, adipose tissue and amnion fluid and were extracted from different species including both human, mice, rats, rabbits, pigs, and lambs. Especially, the studies investigating MSCs in regards to tissue engineering differed considerably in methods and design and it is thus difficult to integrate the results. Furthermore, only two of the eleven studies included in this review reported randomization and only four studies reported blinding when analyses were performed. This is important to bear in mind in regards to potential biases. The studies also differed considerable in regards to the time MSCs were administered as compared to the intervention the animals were exposed to.

As stenosis of the airways is a process characterized by fibrotic wound healing [5] and altered inflammation [6] it is relevant to see if alteration of these factors using MSCs would adjust the development of stenosis. Inhibiting the healing process by inhibiting inflammation and fibrosis would possibly prevent scar tissue formation and prevent a reduced cross-sectional area of the airway. However, the consequence of impaired healing has not yet been investigated.

Another modality of treating severe stenosis with a significant narrowing of the airways, or a long-segment airway stenosis is by creating a graft to substitute the narrow area of the airway. A graft could be helpful in treating both acquired and congenital stenosis. The majority of the studies agreed upon the effect of MSCs in increasing epithelialization. Go, T. *et al*. showed the importance of epithelialization to avoid infections and to create a functional graft. When dealing with laryngotracheal stenosis it is essential to increase the cross-section area and to construction of a functional graft that can expand and widen the airway. The results on the effect of MSCs to aid in increasing the cross-section area were not conclusive.

Mendez JJ. *et al*. [21] concluded that MSCs derived from adipose tissue would be more beneficial than MSCs derived from BM when constructing a patch-type scaffold. Still a lot remains to be investigated such as the possible improved effects of MSCs derived from adipose tissue and if MSCs from adipose tissue is more beneficial in treating subglottic stenosis.

So far no studies on the effect of stem cells and their effect in the airways have been conducted in human trials. Animal studies are associated with a great deal of methodological problems that must be taken into account before the study can be transferred to humans. Methodological problems of animal experiments include difference and variation of metabolic pathways between disparate animal species. Furthermore, variations in dosing regime between human and animals exists as well as variability in methods of randomization, nuances in laboratory technique, and variability in the choice of comparison. [24,25]

This systemic review of the existing animal experiments do represent an important step towards a human trial. [24,25]

In conclusion there are various effects of MSCs on the airways with consistent responses towards inflammation, whereas the effects on fibrosis and tissue repair were contradictory possibly related to methodological differences. Thus it is difficult to summarize the findings into a coherent conclusion. The presented results suggest that MSCs have potential in treating laryngotracheal stenosis but many issues remain to be investigated. Altogether, however, we find that the above-mentioned findings are encouraging for conducting a clinical study.

## Supporting information

S1 Fig PRISMA-flowchart. (DOC)

S1 File PRISMA-checklist. (DOC)
