# Supplementary material for: Mesenchymal stem cell therapy for laryngotracheal stenosis: A systematic review of preclinical studies
Source: PLoS One. 2017 Sep 21;12(9):e0185283. doi: 10.1371/journal.pone.0185283 (PMC5608394; doi:10.1371/journal.pone.0185283)
Supplement: S1 Fig — (DOC) [file pone.0185283.s001.doc]

**PRISMA-flowchart**

**Screening**

**Included**

**Eligibility**

**Identification**

Records identified through database searching
(n = 251)

Additional records identified through other sources
(n = 0)

Records after duplicates removed
(n = 228)

Records screened
(n = 228)

Records excluded
(n = 197)

Full-text articles assessed for eligibility
(n = 31)

Full-text articles excluded, with reasons
(n = 20)

Studies included in qualitative synthesis
(n = 11)

Studies included in quantitative synthesis (meta-analysis)
(n = 11)
